# Effects of steroid therapies to severe streptococcal pneumoniae that required mechanical ventilation

**DOI:** 10.1186/cc12076

**Published:** 2013-03-19

**Authors:** T Nagura, T Ikeda, K Ikeda, T Ueno, S Suda

**Affiliations:** 1 Tokyo Medical University, Hachioji Medical Center, Tokyo, Japan

## Introduction

Streptococcal pneumonia remains the most common cause of community-acquired pneumonia (CAP), bacterial meningitis and bacteremia. Severe pneumonia caused by streptococcal pneumonia frequently exists in the emergency room or ICU. We performed this study to evaluate the effect of steroid therapy for severe streptococcal pneumonia patients with mechanical ventilation retrospectively.

## Methods

We enrolled 13 adults of streptococcal pneumonia patients who required mechanical ventilation. Seven of 13 patients (S group) were administered with steroid (hydrocortisone 200 to 3003 mg/day), and the remaining six patients received no steroid therapy (NS group). As the conventional therapies, mechanical ventilation was commenced when a patient's PaO_2_/FiO_2 _showed less than 200 or they clinically complained of being short of breath. All patients received appropriate fluid therapies, vasoactive agents and blood transfusion according to the protocol of early goal-directed therapy in the Surviving Sepsis Campaign Guidelines 2008, and also were treated with antibiotics, immunoglobulins (5 g/day for 3 days) and sivelestat sodium hydrate (4.83 mg/kg/day for 73days).

## Results

The APACHE scores in the S group and NS group were 27 ± 10 and 23 ± 4, Sequential Organ Failure Assessment scores were 8 ± 4 and 7 ± 3, respectively. These scores showed no significant difference between the groups. Procalcitonin (PCT) in the S and NS groups was 20.7 ± 21.7 and 45.0 ± 47.7 ng/ml, respectively, and there was no significant difference between the groups. PCT declined significantly in both groups. PaO_2_/FiO_2 _of the NS group was significantly higher than the S group on ICU admission and 4 days after admission, but no significant difference on 7 days after ICU admission. IL-6 of the NS group declined significantly after ICU admission, and the S group also tended to decline.

## Conclusion

Steroid therapy for severe streptococcal pneumonia patients with mechanical ventilation may have a potential to maintain oxygenation of the lung, but no significant effects on changes of inflammatory markers (IL-6, CRP).

